# Integrated straw return and biostimulant application improve soil nitrogen supply and grain yield-quality synergy in winter wheat under mild saline-alkaline stress

**DOI:** 10.3389/fpls.2026.1811884

**Published:** 2026-04-17

**Authors:** Qi Xu, Wenda Du, Changkun Ma, Quanjiu Wang

**Affiliations:** 1 State Key Laboratory of Eco-Hydraulics in Northwest Arid Region, Xi’an University of Technology, Xi’an, China; 2 Institute of Arid Region Ecological Water Conservancy, Xi’an University of Technology, Xi’an, China

**Keywords:** nitrogen supply, plant biostimulant, saline-alkaline stress, straw management, wheat productivity

## Abstract

**Introduction:**

Saline-alkaline soils pose significant constraints to sustainable wheat production through impaired nutrient cycling and reduced crop productivity. While straw return and biostimulants are recognized management strategies, their combined effects on soil nutrient dynamics and yield-quality relationships remain poorly understood.

**Methods:**

A two-year field experiment was conducted under mildly saline-alkaline conditions with four treatments: control (CK), straw return (SR), biostimulant foliar application (BS), and integrated straw return with biostimulant (SR+BS). Soil biochemical properties, enzyme activities, crop physiological parameters, yield components, and grain quality were evaluated.

**Results:**

The SR+BS treatment significantly enhanced soil organic carbon (28.3%) and total nitrogen (19.6%) compared to the control. Soil enzyme activities increased markedly, with sucrase, urease, alkaline phosphatase, and catalase showing increases of 67.9%, 32.1%, 47.6%, and 44.5%, respectively. Soil inorganic nitrogen availability was substantially enhanced, with NH_4_^+^-N and NO_3_^−^-N increasing by 47.7% and 39.8%. These improvements were associated with enhanced crop performance: leaf area index and dry matter accumulation increased by 37.5% and 49.1%, respectively. Grain yield reached 8770 kg ha^−1^ (a 40.8% increase), while grain protein content reached 16.4% (a 25.2% increase), demonstrating improved yield-quality relationships under stress conditions.

**Discussion:**

These findings indicate that integrated straw return with biostimulant application drives a cascade-like response — from enhanced soil carbon-nitrogen supply through improved physiological processes to increased crop productivity. This integrated strategy offers a promising and sustainable intensification approach for wheat production in saline-alkaline agroecosystems.

## 1 Introduction

Global food security faces unprecedented challenges from the confluence of population growth, climate change, and land degradation. The world population is projected to reach 9.7 billion by 2050, necessitating a 70% increase in food production (FAO, 2011; Tilman et al., 2011). This challenge is particularly acute in marginal lands, where soil constraints limit agricultural productivity. Among these, saline-alkaline soils represent a critical but underutilized resource, covering approximately 950 million hectares globally (FAO, 2021). China alone possesses 100 million hectares of saline-alkaline land, of which 36% is classified as mildly saline-alkaline (electrical conductivity 2–4 dS m^−1^, pH 7.5-8.5), representing significant potential for agricultural development following appropriate amelioration strategies (Munns and Gilliham, 2015).

Wheat (Triticum aestivum L.), as one of the three major global cereal crops providing 20% of calories and protein for human consumption, exhibits particular sensitivity to saline-alkaline stress (Munns and Gilliham, 2015; Hawkesford and Griffiths, 2019). The compound effects of high pH (>8.0) and elevated salt content fundamentally impair plant productivity by disrupting communication both between organs and among cell types within organs. At the cellular level, osmotic stress triggers ABA-mediated closure of stomatal guard cells, thereby restricting CO_2_ availability to mesophyll cells for photosynthesis, while concurrently impairing water and nutrient absorption in root cortical and pericycle cells (Munns and Tester, 2008; Julkowska and Testerink, 2015). Ionic toxicity arising from Na^+^ and Cl^−^ accumulation disrupts ion loading into xylem vessels at the pericycle and interferes with long-distance root-to-shoot hydraulic and chemical signaling, while nutritional imbalances—particularly for K^+^ and Ca^2+^—compromise turgor regulation and membrane integrity across multiple cell types (Hasegawa et al., 2000). Reactive oxygen species accumulate as a downstream consequence of these metabolic imbalances within and between cell types, and the resulting oxidative damage further compromises mesophyll cell ultrastructure and enzymatic function. These disruptions to inter-organ and inter-cellular communication collectively impair carbon-nitrogen metabolic processes, affecting photosynthesis, nitrogen assimilation, and carbohydrate partitioning, ultimately resulting in 20–50% yield reductions (Zhang et al., 2021; Ji et al., 2022). These stresses severely impair carbon-nitrogen metabolic processes, affecting photosynthesis, nitrogen assimilation, and carbohydrate partitioning, ultimately resulting in 20-50% yield reductions (Amna et al., 2019). Moreover, the characteristic yield-quality tradeoff in wheat becomes more pronounced under stress conditions, as plants prioritize survival over grain filling and protein accumulation (Zheng et al., 2022).

Straw returning has emerged as a fundamental conservation practice for sustainable intensification of agricultural systems. China produces approximately 900 million tons of crop straw annually, with wheat and maize contributing 40% of total production (NBSC, 2020; Zhang et al., 2024). Straw contains 40-45% cellulose, 25-30% hemicellulose, and 15-20% lignin, which upon decomposition release essential nutrients and increase soil organic carbon by 15-30% under continuous application (Zhao et al., 2018; Chen et al., 2017; Islam et al., 2023). However, straw returning in saline-alkaline soils faces unique challenges that limit its effectiveness. The high C:N ratio (60-80:1) of wheat straw induces microbial nitrogen immobilization during initial decomposition phases, potentially exacerbating nitrogen deficiency in already nutrient-limited soils (Fan et al., 2024). Additionally, alkaline conditions (pH >8.0) suppress the activity of lignocellulolytic enzymes and reduce microbial diversity, resulting in decomposition rates 30-40% slower than in neutral soils (Mu et al., 2025). These constraints necessitate complementary strategies to optimize straw utilization efficiency in saline-alkaline agroecosystems (Huang et al., 2021; Shen et al., 2024).

Plant biostimulants represent an innovative category of agricultural inputs that, when applied to plants or the rhizosphere in small quantities, enhance nutrient uptake efficiency, abiotic stress tolerance, and crop quality traits independent of their nutrient content (du Jardin, 2015; EU Regulation, 2019/1009; Yakhin et al., 2017). The multi-component biostimulant employed in this study integrates complementary bioactive substances with distinct but synergistic modes of action. Glutamic acid (5% w/v) serves as a precursor for chlorophyll synthesis and acts as a signaling molecule in nitrogen metabolism, while proline (3% w/v) functions as an osmoprotectant and ROS scavenger under stress conditions (Arslan et al., 2021). Humic acid (10% w/v) enhances cation exchange capacity, chelates micronutrients, and stimulates root plasma membrane H^+^-ATPase activity, thereby improving nutrient acquisition (Canellas et al., 2015; Pathak et al., 2024). Bacillus subtilis (10^8^ CFU/mL), as a plant growth-promoting rhizobacterium, produces extracellular enzymes (cellulases, proteases, phosphatases) that accelerate organic matter decomposition and enhances stress tolerance through induced systemic resistance pathways (Hashem et al., 2019; Blake et al., 2021; Maslennikova et al., 2023).

Recent evidence suggests that combining organic amendments with biostimulants can generate synergistic effects exceeding the sum of individual applications. Meta-analyses indicate that integrated approaches can increase crop yields by 25-40% while reducing chemical fertilizer requirements by 20-30% (Rouphael and Colla, 2020; Calvo et al., 2014). The mechanisms underlying these synergies involve: (i) biostimulant-mediated acceleration of straw decomposition through enhanced enzyme production, (ii) improved synchronization between nutrient release from straw and crop demand, (iii) activation of plant stress-responsive pathways that optimize resource allocation, and (iv) stimulation of beneficial soil microbiota that facilitate nutrient cycling (Bulgari et al., 2019; Bezuglova et al., 2019; Danish and Zafar-ul-Hye, 2019). However, these interactions remain poorly characterized in saline-alkaline soils, where altered soil chemistry and microbiology may modify biostimulant efficacy (Aijaz et al., 2024; Kusale et al., 2021).

Based on the synergistic potential of straw returning and biostimulant application, we hypothesize that their combined use will generate beneficial effects in mildly saline-alkaline soils: (1) Biostimulants will accelerate straw decomposition and nutrient mineralization, alleviating the nitrogen immobilization period and enhancing soil carbon-nitrogen cycling; (2) The improved soil environment will enhance soil enzyme activities and nitrogen availability during critical growth stages; (3) These soil-plant system improvements will be associated with improved crop physiological performance, ultimately improving yield-quality relationships by achieving increases in both grain yield and protein content. This study aims to elucidate these mechanisms through a two-year field experiment, providing both theoretical understanding and practical strategies for sustainable wheat production in saline-alkaline agroecosystems.

## 2 Materials and methods

### 2.1 Experimental site description

**Figure 1:**
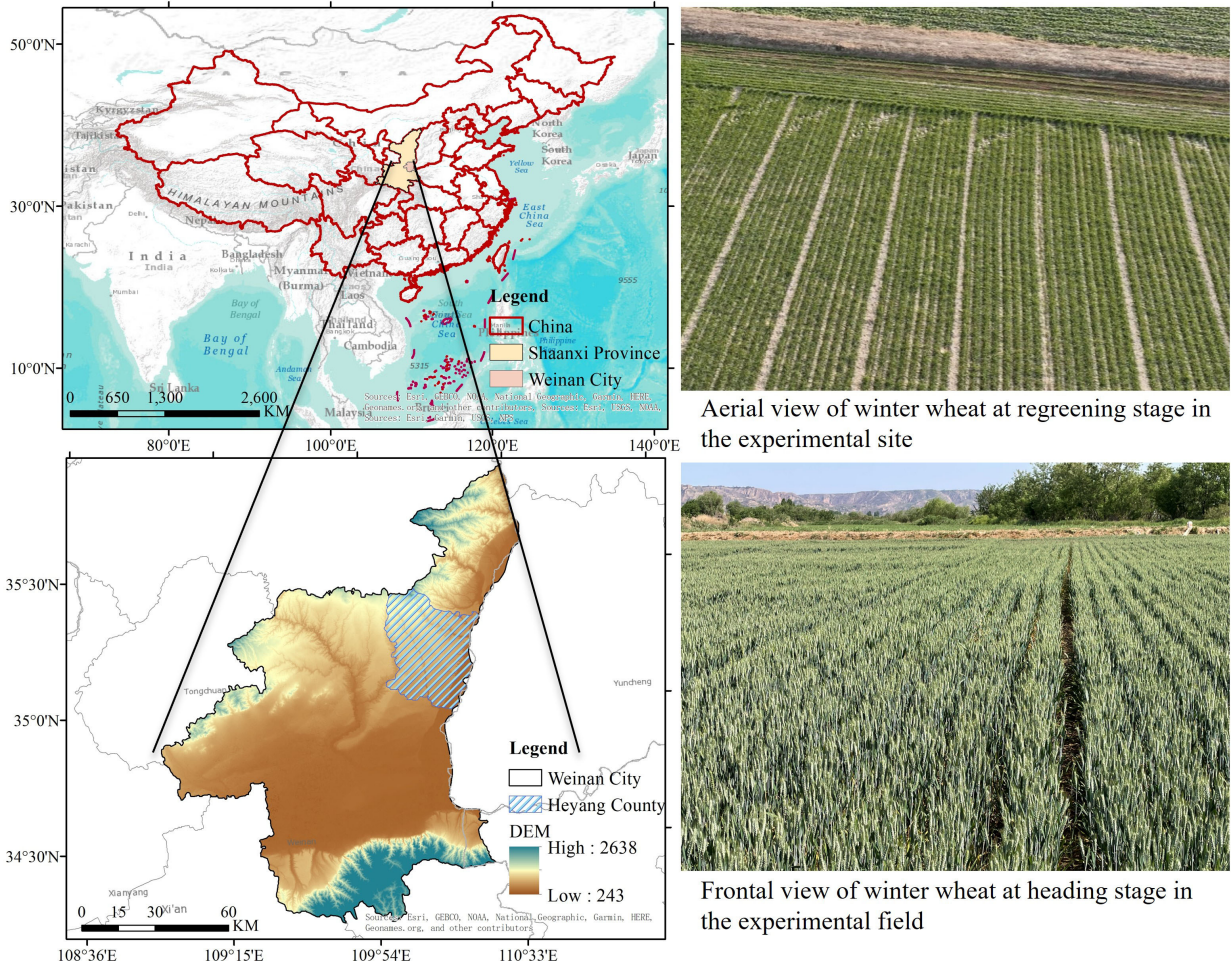
Geographical location of the experimental site and aerial and frontal views of winter wheat.

Field experiments were conducted during two consecutive wheat growing seasons (2023–2024 and 2024-2025) at the Agricultural Experimental Station in Heyang County, Weinan City, Shaanxi Province, China (35°14′40″N, 110°9′24″E, 365 m elevation, **Figure 1**). The region experiences a warm temperate continental monsoon climate with mean annual temperature of 11.5 °C, frost-free period of 197 days, annual sunshine duration of 2858.8 hours, and accumulated temperature ≥0 °C of 4091.8 °C. Long-term average precipitation is 528.2 mm, concentrated mainly during July-September. During the experimental period, total precipitation during the 2023–2024 and 2024–2025 growing seasons was 260.5 mm and 295.4 mm, respectively.

The experimental site soil is classified as mildly saline-alkaline sandy loam, developed from Yellow River alluvial deposits. Pre-sowing soil analysis of the 0–30 cm layer revealed: pH 8.32, electrical conductivity 2.84 dS m^−1^, organic matter 19.8 g kg^−1^, total nitrogen 1.01 g kg^−1^, available nitrogen 78.3 mg kg^−1^, available phosphorus 18.3 mg kg^−1^, available potassium 101.2 mg kg^−1^, cation exchange capacity 12.6 cmol kg^−1^, and exchangeable sodium percentage 8.7%. Soil bulk density was 1.35 g cm^−3^, field capacity 22.3%, and wilting point 9.8%. The previous crop was maize with complete straw removal.

### 2.2 Experimental design

The experiment employed a randomized complete block design with four treatments and three replications, totaling 12 plots. Each plot measured 90 m^2^ (10 m × 9 m) with 2 m protective rows between plots. To ensure fair comparison and account for nitrogen immobilization during straw decomposition, all treatments received equal mineral fertilizer nitrogen inputs through a compensatory design. Treatments included: (1) Control (CK): conventional fertilization management, straw removed, water foliar spray; (2) Straw returning (SR): wheat straw incorporation plus conventional fertilization, water foliar spray; (3) Biostimulant (BS): conventional fertilization, straw removed, biostimulant foliar application at key growth stages; (4) Combined treatment (SR+BS): straw returning plus biostimulant foliar application plus conventional fertilization.

For straw returning treatments, previous season wheat straw was applied at 4500 kg ha^−1^ (based on local wheat straw yield). Straw was mechanically chopped to 2–3 cm lengths, uniformly spread on the soil surface before sowing, and immediately incorporated into the 0–20 cm soil layer using rotary tillage. Based on established nitrogen immobilization coefficients for wheat straw (Chen et al., 2017), an additional 13.8 kg N ha^−1^ was applied to all treatments to maintain nitrogen availability equivalence, ensuring that observed differences were due to treatment effects rather than nitrogen limitation.

The composite biostimulant was developed by Xi’an University of Technology, containing glutamic acid 5 g L^−1^, proline 3 g L^−1^, humic acid 10 g L^−1^, Bacillus subtilis 1×10^8^ CFU mL^−1^, and trace elements (Fe, Zn, B). Foliar application was performed at jointing and grain-filling stages, with application rate of 3 L ha^−1^ diluted to 450 L ha^−1^. Applications were conducted during windless mornings or evenings to ensure optimal leaf absorption. Control and SR treatments received equivalent water volumes.

### 2.3 Crop management

Winter wheat cultivar Jimai 22 was used, selected for its high yield potential, quality traits, and disease resistance, suitable for the Yellow-Huai River wheat production region. Sowing rate was 225 kg ha^−1^, row spacing 20 cm, and sowing depth 3–4 cm. Sowing dates were October 8, 2023 and October 10, 2024, with harvesting on June 1, 2024 and June 3, 2025, respectively.

Fertilization followed locally optimized recommendations. All treatments received basal application of compound fertilizer 750 kg ha^−1^ (N-P_2_O_5_-K_2_O: 15-15-15), equivalent to 112.5 kg ha^−1^ each of N, P_2_O_5_, and K_2_O. Topdressing of urea 150 kg ha^−1^ (69 kg N ha^−1^) was applied at jointing stage. Considering nitrogen requirements for straw decomposition, all treatments received additional urea 30 kg ha^−1^ (13.8 kg N ha^−1^), ensuring total nitrogen application of 195.3 kg ha^−1^. All phosphorus and potassium fertilizers were applied basally.

Irrigation employed border irrigation with flow meters controlling water amounts. Four irrigations were applied during the growing season at wintering, jointing, heading, and grain-filling stages, each supplying 40 mm. Pest and disease management followed local plant protection recommendations to ensure treatment effects were not compromised. Weeds were controlled manually.

### 2.4 Sampling and measurements

#### 2.4.1 Soil sampling and analysis

Soil samples were collected before sowing, at jointing, flowering, grain-filling stages, and after harvest. Five sampling points per plot were arranged in an S-pattern using a 5-cm diameter soil auger for 0–20 cm depth, composited into one sample. Fresh soil was sieved through 2 mm mesh; one portion was immediately analyzed for moisture content, ammonium nitrogen, nitrate nitrogen, and soil enzyme activities, while another portion was air-dried for other physicochemical analyses.

Basic soil properties were determined following standard methods. Soil pH was measured potentiometrically (water:soil ratio 2.5:1), electrical conductivity using a conductivity meter (water:soil ratio 5:1). Organic carbon was determined by potassium dichromate oxidation, total nitrogen by Kjeldahl digestion. Available nitrogen was measured by alkaline hydrolysis diffusion, available phosphorus by sodium bicarbonate extraction-molybdenum antimony colorimetry, and available potassium by ammonium acetate extraction-flame photometry. Ammonium nitrogen (NH_4_^+^-N) and nitrate nitrogen (NO_3_^−^-N) were extracted with 2 M KCl solution and determined using a continuous flow analyzer (AA3 HR AutoAnalyzer, SEAL Analytical GmbH, Norderstedt, Germany) by the indophenol blue colorimetric method and the cadmium reduction colorimetric method, respectively.

Soil enzyme activities were determined following Guan et al. (1986). Sucrase activity was measured using 3,5-dinitrosalicylic acid colorimetry, expressed as mg glucose kg^−1^ soil h^−1^. Urease activity was determined by phenol-sodium hypochlorite colorimetry, expressed as mg NH_4_^+^ kg^−1^ soil h^−1^. Alkaline phosphatase activity was measured using disodium phenyl phosphate colorimetry, expressed as μg phenol kg^−1^ soil h^−1^. Catalase activity was determined by potassium permanganate titration, expressed as mL 0.1 mol L^−1^ KMnO_4_ kg^−1^ soil h^−1^.

#### 2.4.2 Plant sampling and analysis

Plant samples were collected at major growth stages including jointing, flowering, grain-filling, and maturity. One square meter of uniform growth was harvested from each plot at ground level, separated by organs, and bagged. Samples were oven-dried at 105 °C for 30 minutes, then at 75 °C to constant weight for dry matter determination. Dried samples were ground through 0.5 mm sieves for nutrient content analysis.

Leaf area index was determined using the length-width coefficient method. At greening, jointing, flowering, and grain-filling stages, 10 representative plants per plot were selected to measure all green leaf lengths and maximum widths. Leaf area equals length × width × 0.78 (wheat leaf area coefficient). Leaf area index equals total leaf area per unit ground area.

Chlorophyll content was determined by ethanol extraction. Fresh leaves (0.1 g) were extracted in 10 mL 95% ethanol for 24 hours in darkness, with absorbance measured at 665 and 649 nm. Chlorophyll a content (mg g^−1^) = 13.95×A_665_ - 6.88×A_649_; Chlorophyll b content (mg g^−1^) = 24.96×A_649_ - 7.32×A_665_.

Plant nutrient contents were determined after H_2_SO_4_-H_2_O_2_ digestion. Plant samples (0.2 g) were digested with concentrated sulfuric acid and hydrogen peroxide until clear, then diluted for analysis. Total nitrogen was determined by Kjeldahl method or continuous flow analyzer, total phosphorus by molybdenum antimony colorimetry, and total potassium by flame photometry.

### 2.5 Yield and quality assessment

At maturity, three 1-m^2^ quadrats per plot were harvested for yield determination. Spike number per unit area was counted, with 20 randomly selected spikes for kernel number determination. After threshing and natural air-drying, thousand-kernel weight and grain yield were measured, with moisture content adjusted to 13%. Harvest index was calculated as grain yield divided by aboveground biomass.

Grain quality was assessed after one-month storage post-harvest when moisture stabilized. Protein content, wet gluten content, gluten index, and sedimentation value were determined using Fourier transform near-infrared analyzer (MATRZX-1, Germany). Dough rheological properties including water absorption, development time, stability time, and degree of softening were measured using a Farinograph (Brabender, Germany). Starch content was determined by polarimetry. All quality parameters followed national standard methods.

### 2.6 Statistical analysis

Data were preliminarily processed using Microsoft Excel 2019 for means and standard errors. Statistical analyses employed SPSS 26.0. Treatment differences were assessed by one-way ANOVA with Duncan’s multiple range test for mean separation at P<0.05 significance level. Two-way ANOVA evaluated effects of year, treatment, and their interactions. Year × treatment interactions were non-significant (P>0.05) for most parameters, indicating consistent treatment effects across years.

Correlation analysis used Pearson correlation coefficients. Principal component analysis assessed relative contributions of different indicators. Structural equation modeling (SEM) using AMOS 24.0 analyzed causal relationships among soil-plant-yield factors. Model fit was evaluated by chi-square/degrees of freedom ratio (χ^2^/df<3), goodness-of-fit index (GFI>0.9), comparative fit index (CFI>0.9), and root mean square error of approximation (RMSEA<0.08).

Entropy-weighted TOPSIS method provided comprehensive treatment evaluation. Indicators were standardized, information entropy and weights calculated, weighted normalized matrix constructed, positive and negative ideal solutions determined, distances and relative proximity calculated, and treatments ranked. Figures were created using Origin 2021 and GraphPad Prism 8.0.

## 3 Results

### 3.1 Meteorological conditions and soil carbon-nitrogen status

Meteorological conditions during the experimental period favored wheat growth, with distinct seasonal patterns between the two growing seasons (**Table 1**). Total precipitation during the 2024–2025 growing season (295.4 mm) exceeded that of 2023-2024 (260.5 mm) by 13.3%, with rainfall concentrated primarily during jointing and grain-filling stages. Temperature regimes remained comparable between years, although earlier spring warming in 2024–2025 advanced wheat greening and tillering by approximately one week. The precipitation pattern exhibited typical seasonal distribution, with winter drought conditions transitioning to spring rainfall that coincided with critical growth phases.

**Table 1:** Monthly meteorological conditions during the winter wheat growing seasons.

Year	Month	Mean monthly temperature (°C)	Total monthly precipitation (mm)	Mean monthly relative humidity (%)	Mean monthly wind speed (m s^−1^)	Total monthly evaporation(mm)
2023-2024	Oct	15.1	32.4	60	2.3	88
	Nov	8.7	20.6	57	2.2	66
	Dec	2.5	6.3	45	2.4	41
	Jan	0.4	5.2	47	2.3	36
	Feb	3.9	8.7	50	2.5	46
	Mar	10.1	22.4	52	2.6	87
	Apr	15.6	34.8	54	2.6	118
	May	20.8	64.3	56	2.5	147
	Jun	25.4	65.8	48	2.4	168
2024-2025	Oct	14.7	36.2	61	2.3	90
	Nov	8.3	24.5	58	2.3	68
	Dec	2.2	7.6	46	2.4	42
	Jan	0.5	6.8	48	2.3	37
	Feb	4.1	10.4	51	2.5	48
	Mar	10.5	27.3	53	2.6	90
	Apr	16.0	41.7	55	2.6	121
	May	21.1	69.8	57	2.5	150
	Jun	25.6	71.1	49	2.4	170

Soil organic carbon and total nitrogen contents varied differentially among treatments (**Table 2**). The SR+BS treatment achieved the highest SOC levels, reaching 23.8 and 23.9 g kg^−1^ in 2023–2024 and 2024-2025, respectively, representing increases of 28.3% and 27.1% over control. These enhancements significantly exceeded single treatments, with SR alone increasing SOC by 14.0-15.9% and BS alone by 6.5-8.0%. Total nitrogen followed similar patterns, with SR+BS achieving 1.03 and 1.01 g kg^−1^, corresponding to 21.2% and 17.4% increases over control. The C/N ratio under SR+BS maintained optimal levels (23.2-23.7) for microbial activity, indicating balanced organic matter decomposition and nitrogen cycling.

**Table 2:** Effects of different treatments on soil carbon-nitrogen status and inorganic nitrogen at grain-filling stage.

Year	Treatment	Soil organic carbon (g kg^-1^)	Soil total nitrogen (g kg^-1^)	C/N ratio	NH_4_^+^-N (mg kg^-1^)	NO_3_^−^-N (mg kg^-1^)
2023-2024	CK	18.6 ± 0.8c	0.85 ± 0.03c	21.9 ± 0.2a	12.8 ± 0.9c	25.9 ± 1.6 d
	SR	21.2 ± 1.1b	0.92 ± 0.04b	23.1 ± 1.7a	15.6 ± 1.1 b	29.6 ± 1.8 c
	BS	19.8 ± 0.9bc	0.87 ± 0.03c	22.8 ± 1.8a	14.2 ± 1.0 bc	34.7 ± 2.1 b
	SR+BS	23.8 ± 1.2a	1.03 ± 0.05a	23.2 ± 2.3a	18.9 ± 1.3 a	36.2 ± 2.2 a
2024-2025	CK	18.8 ± 0.9c	0.86 ± 0.04c	21.9 ± 0.7a	13.1 ± 0.8 c	26.4 ± 1.5 d
	SR	21.8 ± 1.0b	0.95 ± 0.04b	23.0 ± 1.9a	16.2 ± 1.0 b	30.8 ± 1.7 c
	BS	20.3 ± 1.1bc	0.89 ± 0.04c	22.9 ± 1.9a	14.8 ± 0.9 bc	35.9 ± 2.0 b
	SR+BS	23.9 ± 1.1a	1.01 ± 0.05a	23.7 ± 1.5a	19.5 ± 1.2 a	37.8 ± 2.1 a
Mean(2023-2025)	CK	18.7 ± 0.8d	0.85 ± 0.03c	21.9 ± 0.5a	12.9 ± 0.8c	26.1 ± 1.5d
	SR	21.5 ± 1.0b	0.93 ± 0.04b	23.0 ± 1.6a	15.9 ± 1.1b	30.2 ± 1.7c
	BS	20.5 ± 1.0c	0.88 ± 0.04c	22.8 ± 1.7a	14.5 ± 0.9b	35.3 ± 2.0b
	SR+BS	23.8 ± 1.0a	1.02 ± 0.05a	23.4 ± 1.8a	19.2 ± 1.2a	37.0 ± 2.1a

Different lowercase letters within the same column indicate significant differences among treatments at P<0.05 according to Duncan’s multiple range test. CK represents control treatment with conventional fertilization and straw removal; SR represents straw returning at 4500 kg ha^−1^ with conventional fertilization; BS represents biostimulant foliar application with conventional fertilization; SR+BS represents combined straw returning and biostimulant application with conventional fertilization. Mean(2023–2025) represents the average values of each indicator across the four treatments during the two-year growing season.

Values are means ± standard error (n=3).

Soil inorganic nitrogen fractions showed distinct patterns across treatments (**Table 2**). NH_4_^+^-N concentrations ranged from 12.8 to 19.5 mg kg^−1^, with SR+BS treatment achieving the highest levels, representing 47.7% and 48.9% increases over control in 2023–2024 and 2024-2025, respectively (P<0.05). This substantial enhancement exceeded the additive effects of individual SR (22.4%) and BS (11.7%) treatments, indicating synergistic stimulation of nitrogen mineralization. NO_3_^−^-N concentrations exhibited greater absolute increases under SR+BS (36.2-37.8 mg kg^−1^), corresponding to 39.8-43.2% increases over control. Notably, the BS treatment showed proportionally greater enhancement in NO_3_^−^-N (34.7 mg kg^−1^, +34.0%) compared to NH_4_^+^-N (+11.7%), suggesting biostimulant-mediated acceleration of nitrification processes.

**Figure 2:**
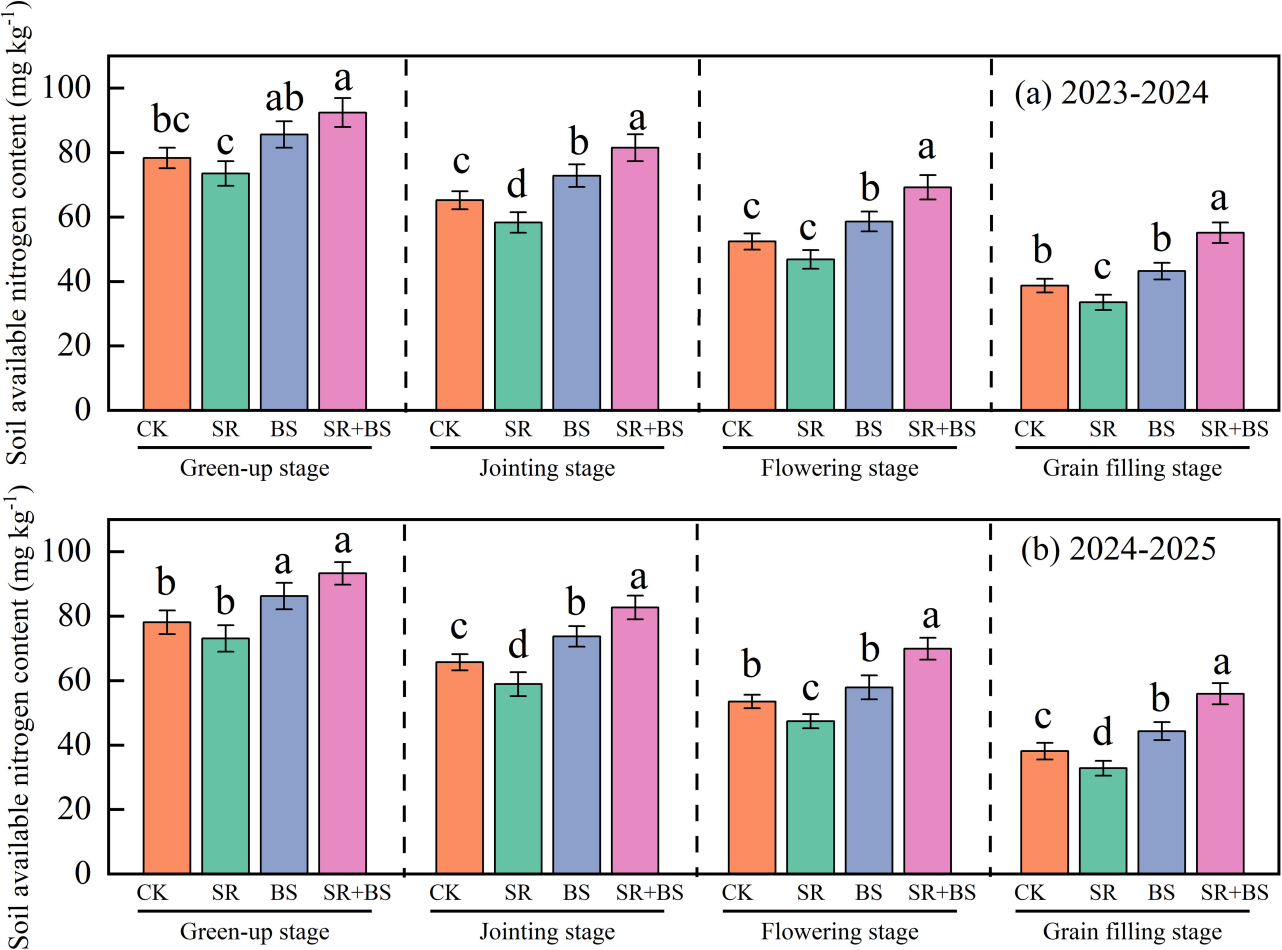
Dynamics of soil available nitrogen content under different treatments. Vertical bars represent standard errors of the mean (n=3). Different letters above bars indicate significant differences among treatments at the same growth stage at P<0.05 according to Duncan’s multiple range test, where letter ‘a’ represents the highest value group and subsequent letters indicate progressively lower values with significant separation. Soil samples were collected from 0–20 cm depth at each growth stage for available nitrogen determination by alkaline hydrolysis diffusion method. Treatment abbreviations as in **Table 2** note.

Temporal dynamics of soil available nitrogen revealed distinct treatment effects on nutrient supply patterns (**Figure 2**). At jointing stage, SR+BS achieved 81.5 mg kg^−1^, significantly exceeding other treatments (P<0.05). Despite progressive decline through the growing season, SR+BS maintained consistently elevated levels. At the critical grain-filling stage, SR+BS sustained 55.1 mg kg^−1^ available nitrogen, 42.3% higher than control. The SR treatment exhibited initial nitrogen immobilization at jointing (58.3 mg kg^−1^), attributed to microbial demand during straw decomposition. However, biostimulant application effectively mitigated this limitation through provision of readily available nitrogen sources.

### 3.2 Soil enzyme activities and temporal dynamics

**Figure 3:**
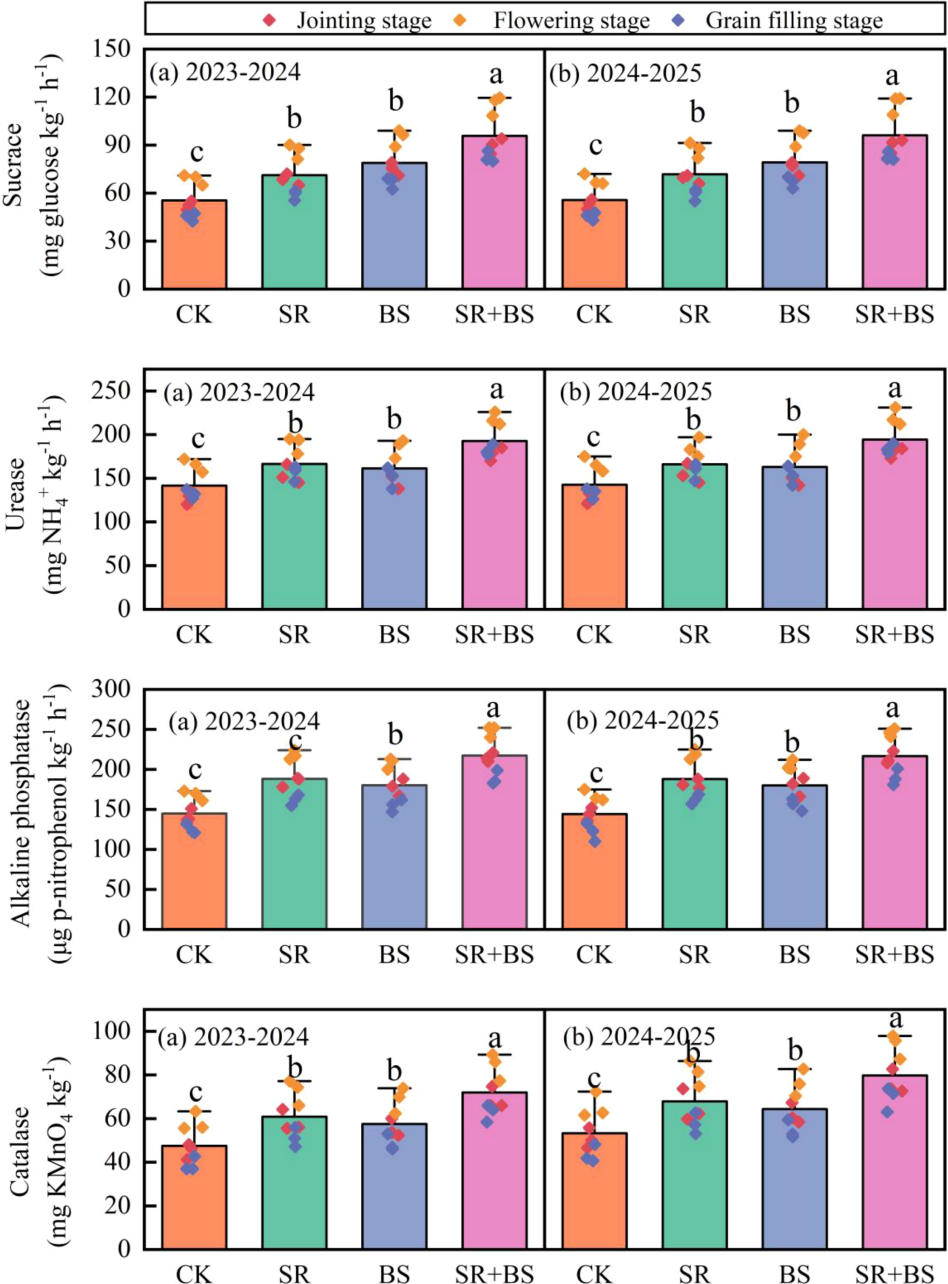
Soil carbon-nitrogen metabolic enzyme activities at different growth stages. Error bars represent standard errors (n=3) and different letters at thesame growth stage indicate significant differences among treatments at P<0.05 based on Duncan’s multiple range test. Treatment abbreviations as in **Table 2** note.

Soil enzyme activities exhibited treatment-specific temporal patterns, with maximum responses occurring at flowering stage (**Figure 3**). Sucrase activity, indicative of carbon cycling intensity, peaked under SR+BS at 115.3 mg glucose kg^−1^ soil h^−1^, representing 1.68-, 1.22-, and 1.33-fold increases over CK, SR, and BS treatments, respectively (P<0.05). This enhancement suggests synergistic interactions between straw-derived carbon substrates and biostimulant-associated microorganisms in promoting carbon transformation.

Urease activity patterns paralleled sucrase dynamics, with SR+BS achieving maximum values of 218 mg NH_4_^+^ kg^−1^ soil h^−1^ at flowering, 32.1% higher than control. While SR alone showed limited urease enhancement (14.5% over control), biostimulant addition substantially amplified this effect, indicating critical roles of beneficial microorganisms in nitrogen cycling. The moderate increase reflects partial inhibition by saline-alkaline conditions (pH 8.3), which biostimulant-associated bacteria effectively ameliorated.

Alkaline phosphatase activity reached 248 μg p-nitrophenol kg^−1^ soil h^−1^ at flowering under SR+BS, significantly exceeding all other treatments (P<0.05). The enzyme maintained high activity due to optimal pH alignment between enzyme function (pH 8.0-10.0) and soil conditions (pH 8.3). Catalase activity increased by 44.5% under SR+BS, reflecting enhanced biological activity and oxidative stress responses. Year-on-year comparisons revealed cumulative effects, with second-year soil enzyme activities exceeding first-year levels by 12-18% for straw-returning treatments.

### 3.3 Plant growth dynamics and biomass accumulation

**Figure 4:**
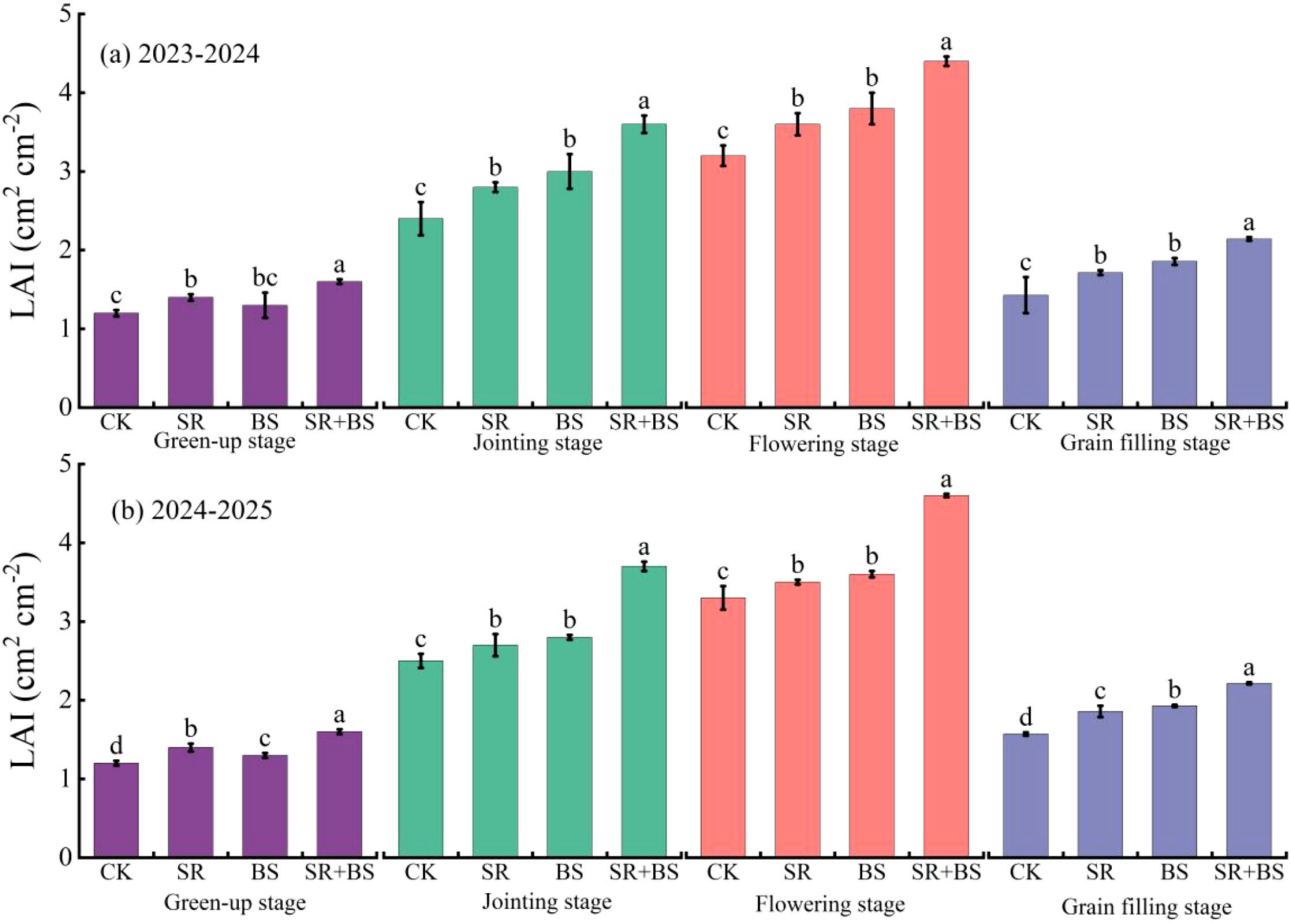
Dynamics of winter wheat leaf area index under different treatments. Values are means of three replicates with vertical bars representing standard errors. Leaf area index was determined using the length-width coefficient method with measurements taken from 10 representative plants per plot at each growth stage. LAI, leaf area index; Treatment abbreviations as in **Table 2** note.

Leaf area index development showed pronounced treatment effects throughout the growing season (**Figure 4**). Maximum LAI at flowering reached 4.4 under SR+BS in both seasons, representing a 37.5% increase over control (P<0.05). Critically, SR+BS delayed leaf senescence, maintaining LAI at 3.0 during grain-filling compared to 2.2 in control. While BS alone improved LAI moderately (18.8% increase), the combined treatment demonstrated superior canopy architecture maintenance.

**Figure 5:**
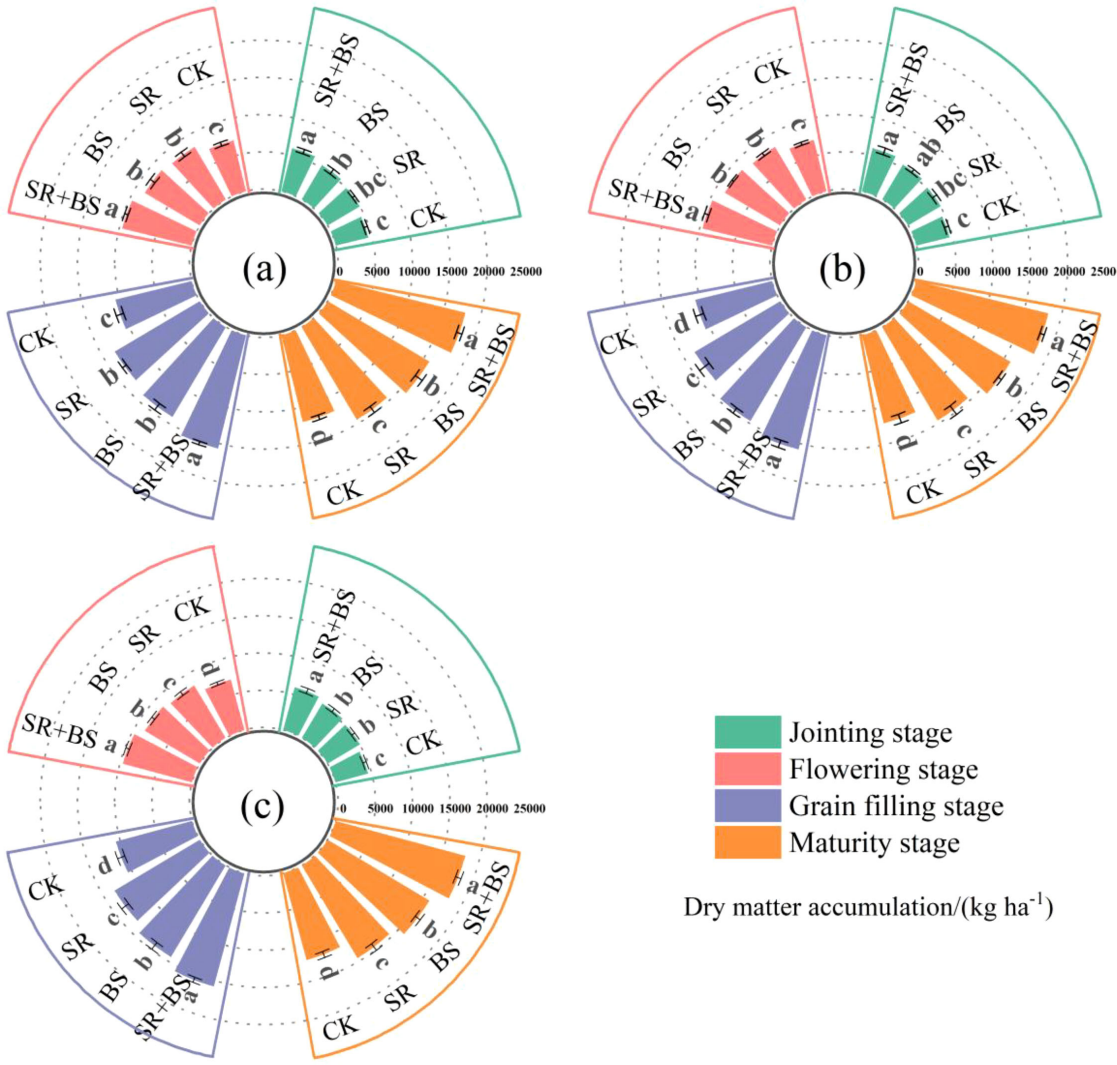
Dynamics of dry matter accumulation in winter wheat under different treatments. Data points represent the mean of three replicates, with vertical bars indicating standard errors. **(a)** represents the 2023–2024 growing season, **(b)** represents the 2024–2025 growing season, and **(c)** represents the average of the two growing seasons (2023–2025). Treatment abbreviations as in **Table 2** note.

Dry matter accumulation exhibited progressive increases across growth stages, with significant treatment differentiation (**Figure 5**). At jointing stage, treatment differences remained relatively modest, with SR+BS accumulating approximately 4500 kg ha^−1^. Biomass accumulation accelerated substantially during subsequent reproductive phases. By flowering stage, SR+BS reached approximately 9500–10000 kg ha^−1^, establishing clear superiority over other treatments (P<0.05). The divergence among treatments became increasingly pronounced through grain filling, ultimately culminating in distinct final biomass levels at maturity. SR+BS achieved the highest aboveground biomass of 17895 kg ha^−1^ at maturity, significantly exceeding CK treatments by 49.1%, respectively (P<0.05). Second-year results corroborated first-year trends, demonstrating the consistency and reliability of treatment effects on dry matter accumulation dynamics.

### 3.4 Plant nutritional status and photosynthetic capacity

Leaf nutrient concentrations at grain-filling demonstrated comprehensive nutritional enhancement under SR+BS (**Table 3**). Nitrogen, phosphorus, and potassium contents reached 32.7, 3.28, and 25.1 g kg^−1^, representing increases of 45.5%, 45.8%, and 46.5% over control, respectively (P<0.05). These improvements exceeded additive effects of individual treatments, with SR alone achieving 18-22% increases and BS alone 12-15% increases.

**Table 3:** Crop leaf nutrient contents at grain-filling stage under different treatments.

Year	Treatment	Leaf nitrogen content (g kg^-1^)	Leaf phosphorus content (g kg^-1^)	Leaf potassium content (g kg^-1^)
2023-2024	CK	22.4 ± 1.6d	2.25 ± 0.11c	16.8 ± 1.1c
	SR	25.8 ± 1.4c	2.48 ± 0.22bc	19.2 ± 1.0b
	BS	28.3 ± 1.0b	2.78 ± 0.17b	21.5 ± 1.1b
	SR+BS	32.1 ± 1.0a	3.21 ± 0.22a	24.8 ± 1.6a
2024-2025	CK	23.1 ± 1.6d	2.32 ± 0.17c	17.2 ± 1.5c
	SR	26.3 ± 1.7c	2.56 ± 0.12c	19.8 ± 1.7bc
	BS	29.1 ± 1.5b	2.89 ± 0.19b	22.1 ± 1.4b
	SR+BS	33.2 ± 0.9a	3.35 ± 0.09a	25.4 ± 1.9a
Mean(2023-2025)	CK	22.7 ± 1.5d	2.28 ± 0.14d	17.0 ± 1.2d
	SR	26.0 ± 1.4c	2.52 ± 0.16c	19.5 ± 1.3c
	BS	28.7 ± 1.2b	2.83 ± 0.17b	21.8 ± 1.2b
	SR+BS	32.6 ± 1.1a	3.28 ± 0.17a	25.1 ± 1.6a

Different lowercase letters within the same column and year indicate significant differences among treatments at P<0.05 based on Duncan’s multiple range test. Leaf samples were collected from flag leaves at mid-grain-filling stage (20 days after anthesis) for nutrient analysis. Mean(2023–2025) represents the average values of each indicator across the four treatments during the two-year growing season. Treatment abbreviations as in **Table 2** note.

Data are presented as means ± standard error of three replicates.

**Figure 6:**
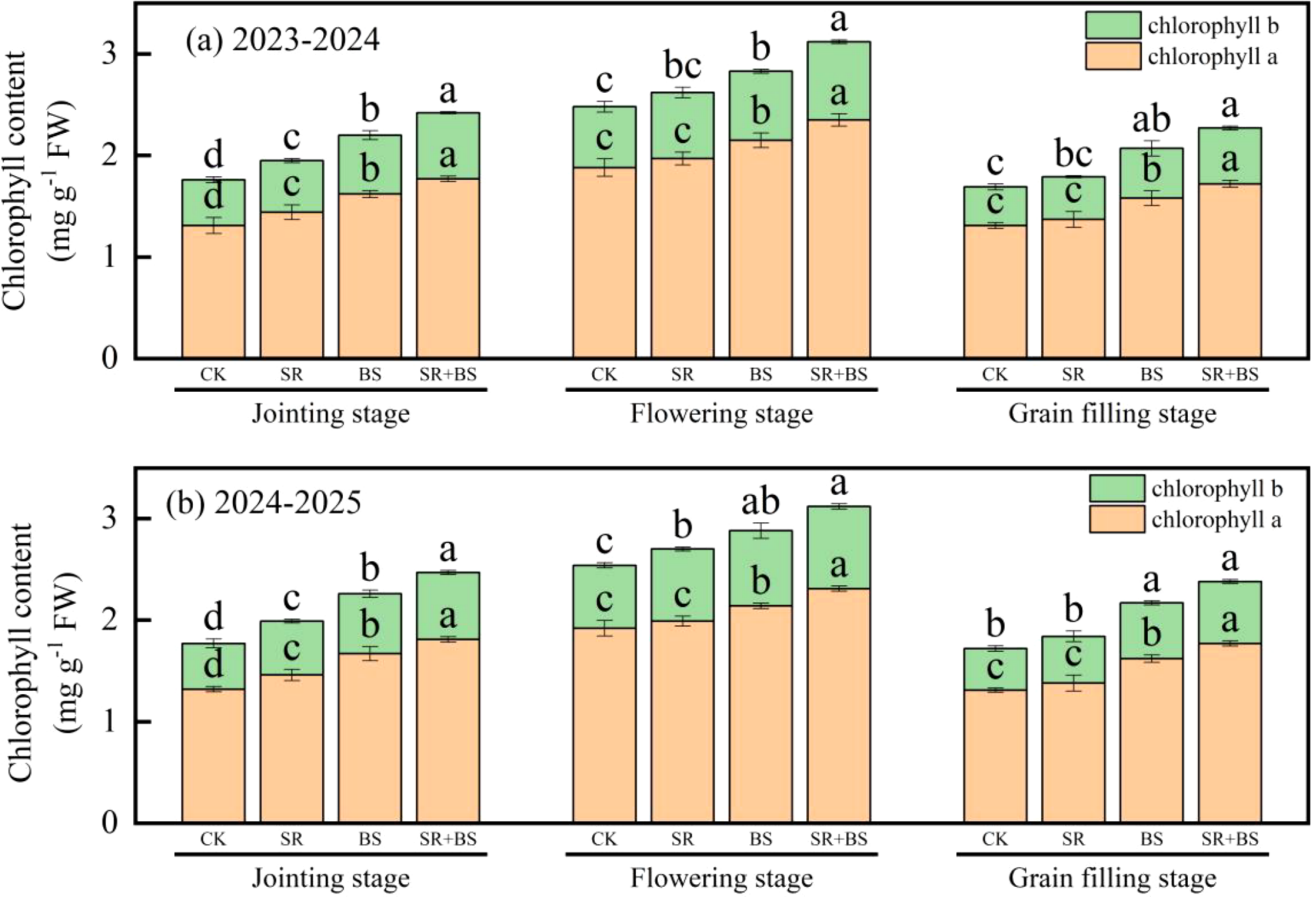
Chlorophyll content changes under different treatments. The figure shows **(a)** Chlorophyll a content and **(b)** Chlorophyll b content expressed as mg g^−1^ fresh weight. Error bars represent standard errors of three replicates. Treatment abbreviations as in **Table 2** note.

Chlorophyll dynamics reflected enhanced photosynthetic capacity under SR+BS treatment (**Figure 6**). At flowering, chlorophyll a and b contents reached 2.35 and 0.77 mg g^−1^, respectively, significantly exceeding other treatments (P<0.05). The chlorophyll a/b ratio maintained optimal values (3.05-3.12), indicating well-balanced light-harvesting complex development.

### 3.5 Yield components and grain quality parameters

Yield component analysis demonstrated that SR+BS achieved yield enhancement through coordinated improvement across multiple traits (**Table 4**). Two-year average grain yield reached 8770 kg ha^−1^ under SR+BS, representing a 40.8% increase over control (P<0.05). This enhancement significantly exceeded individual treatments (SR: 18.0% increase; BS: 29.0% increase). Yield structure analysis revealed modest spike number increase (7.4%), with primary contributions from kernels per spike (17.9% increase) and thousand-kernel weight (11.2% increase). The exceptional yield response, surpassing typical biostimulant effects (15-35%), suggests synergistic stress alleviation mechanisms under saline-alkaline conditions.

**Table 4:** Winter wheat yield and yield components under different treatments (two-year average).

Treatment	Spike numbers (×10^4^ ha^-1^)	Kernel numbers per spike (kernels spike^-1^)	1000-grain weight (g)	Grain yield (kg ha^-1^)	Yield increase rate (%)
CK	498 ± 9 c	34.1 ± 1.3 d	36.7 ± 1.0 d	6230 ± 239 d	–
SR	512 ± 9 bc	37.6 ± 0.6 c	38.2 ± 0.4 c	7354 ± 178 c	18.03
BS	523 ± 12 ab	38.9 ± 0.4 b	39.5 ± 0.4 b	8035 ± 181 b	28.98
SR+BS	535± 5 a	40.2 ± 0.6 a	40.8 ± 0.4 a	8770 ± 388 a	40.76

Different lowercase letters within the same column denote significant differences among treatments at P<0.05 according to Duncan’s multiple range test. Yield components were determined from three 1-m^2^ quadrats per plot at physiological maturity, with grain yield adjusted to 13% moisture content. Treatment abbreviations as in **Table 2** note.

Values represent means ± standard error of six observations (3 replicates × 2 years).

Grain quality parameters showed marked improvement under SR+BS treatment (**Table 5**). Protein content reached 16.4%, a 25.2% increase over control, achieving strong-gluten wheat standards (≥14%). Wet gluten content increased to 35.7%, gluten index improved to 42, and sedimentation value reached 39.8 mL. Starch content maintained at 64.1%, ensuring balanced nutritional composition.

**Table 5:** Winter wheat grain quality parameters under different treatments (two-year average).

Treatment	Protein content (%)	Wet gluten content (%)	Gluten index	Sedimentation value (mL)	Starch content (%)
CK	13.1 ± 0.4 d	28.4 ± 1.1 d	29 ± 2 c	28.8 ± 1.2 d	68.2 ± 0.5 a
SR	14.2 ± 0.4 c	32.7 ± 1.1 c	31 ± 2 c	33.3 ± 1.7 c	66.8 ± 1.2 b
BS	15.1 ± 0.4 b	33.5 ± 1.4 b	39 ± 2 b	37.4 ± 1.5 b	65.4 ± 1.6 c
SR+BS	16.4 ± 0.2 a	35.7 ± 1.0 a	42 ± 2 a	39.8 ± 1.4 a	64.1 ± 0.7 d

Different lowercase letters within the same column indicate significant differences among treatments at P<0.05 based on Duncan’s multiple range test. Quality parameters were analyzed after one-month post-harvest storage when grain moisture content stabilized at approximately 13%. Treatment abbreviations as in **Table 2** note.

Data are means ± standard error of six determinations.

### 3.6 Integrated assessment and mechanistic pathways

Entropy-weighted TOPSIS analysis incorporating indicators revealed comprehensive treatment performance (**Table 6**). SR+BS achieved the highest relative proximity coefficient (0.818), followed by BS (0.662), SR (0.384), and CK (0.161), confirming superior multi-dimensional performance (P<0.05).

**Table 6:** Comprehensive evaluation of treatments based on TOPSIS method.

Treatment	D_i_^+^	D_i_^-^	C_i_	Ranking
CK	0.847	0.163	0.161	4
SR	0.634	0.396	0.384	3
BS	0.342	0.671	0.662	2
SR+BS	0.185	0.832	0.818	1

Di+ represents the distance from the positive ideal solution, Di- represents the distance from the negative ideal solution, and Ci represents the relative proximity coefficient ranging from 0 to 1, with higher values indicating better overall performance. Treatment abbreviations as in **Table 2** note.

The entropy-weighted TOPSIS analysis incorporated indicators including yield components, grain quality parameters, soil properties, soil enzyme activities, and crop-related indicators.

**Figure 7:**
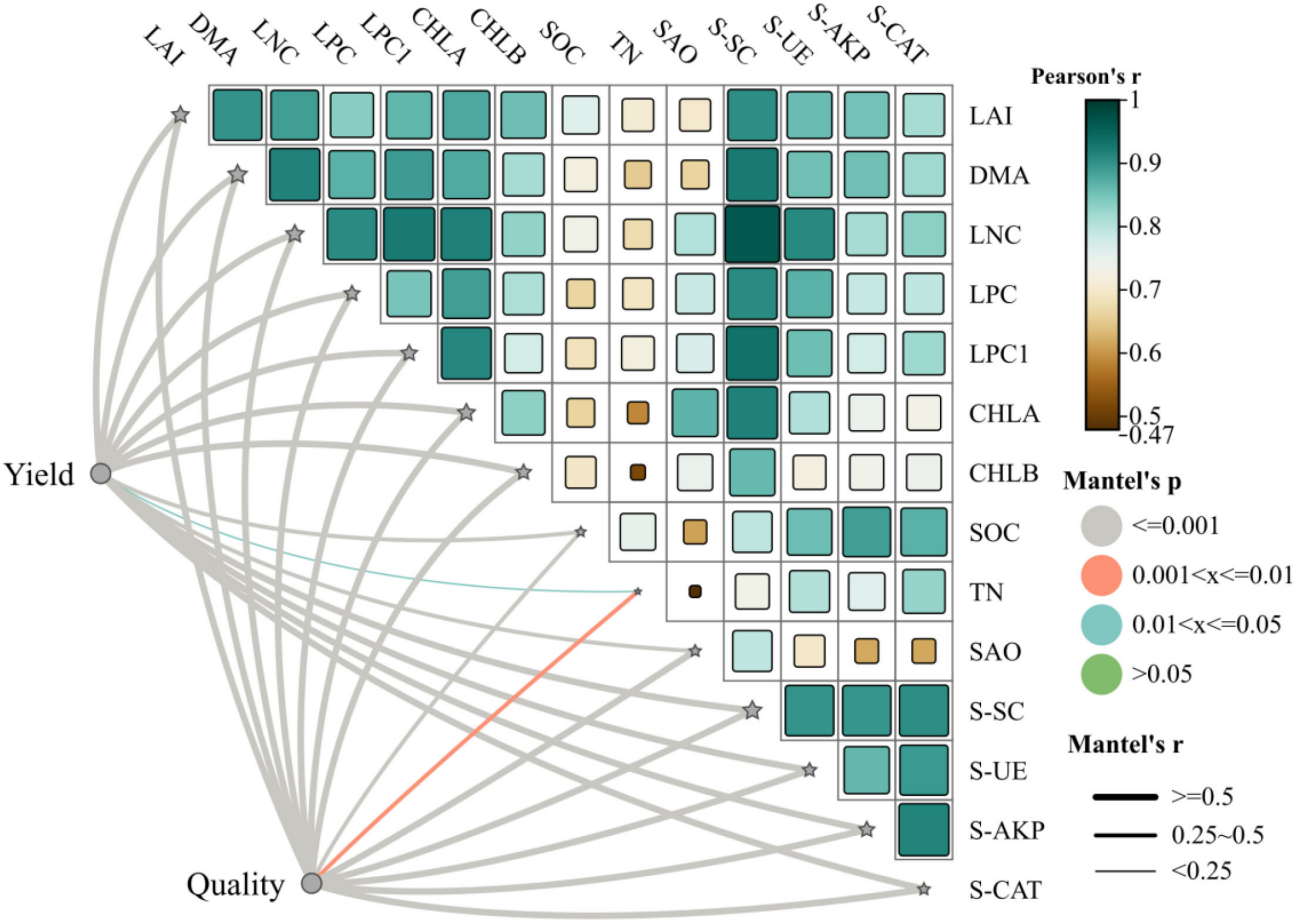
Correlation heatmap between different indicators. The color gradient from dark brown to white to dark cyan represents the range of correlation coefficients from -1 (strong negative correlation) to 0 (no correlation) to +1 (strong positive correlation). LAI, leaf area index; DMA, dry matter accumulation; LNC, leaf nitrogen content; LPC, leaf phosphorus content; LPC1, Leaf potassium content; CHLA, chlorophyll a content; CHLB, chlorophyll b content; SOC, soil organic carbon; TN, soil total nitrogen; SAO, soil available nitrogen; S-SC, soil sucrase activity; S-UE, soil urease activity; S-AKP, soil alkaline phosphatase activity; S-CAT, soil catalase activity.

Correlation analysis elucidated intricate relationships among soil properties, physiological parameters, and production outcomes (**Figure 7**). The correlation heatmap revealed predominantly positive associations among measured indicators, with correlation coefficients ranging from 0.37 to 1.0. Soil organic carbon (SOC) exhibited strong positive correlations with soil enzyme activities, particularly sucrase (S-SC, r>0.8, P<0.01), indicating close coupling between soil carbon status and microbial carbon metabolism. Total nitrogen (TN) showed robust correlations with urease activity (S-UE, r>0.8, P<0.01), reflecting the tight linkage between soil nitrogen pools and enzymatic nitrogen transformation capacity.

**Figure 8:**
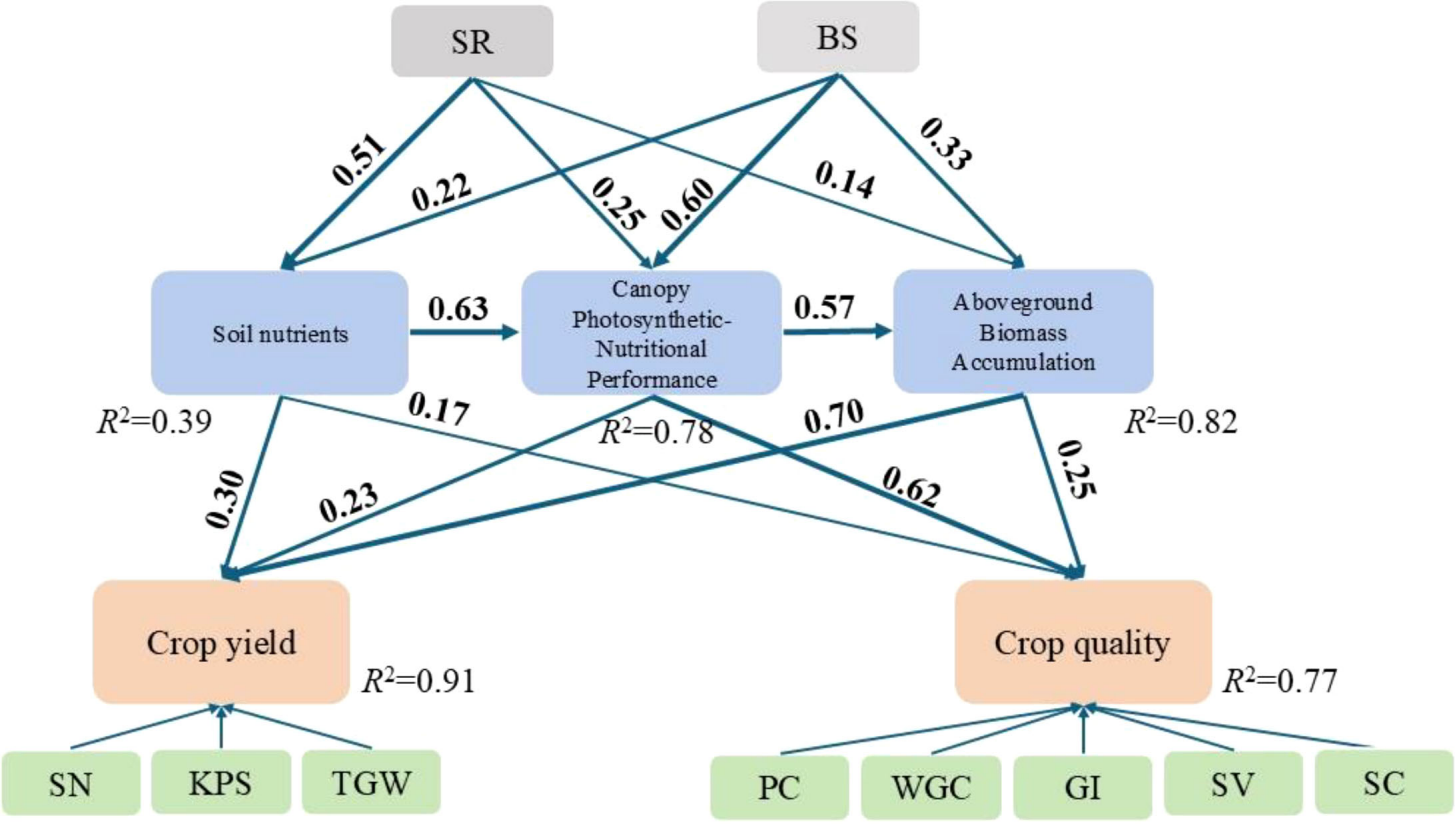
Structural equation model of straw returning and biostimulant effects on wheat production efficiency. Solid lines indicate positive effects with line thickness representing effect magnitude. Numbers adjacent to paths show standardized path coefficients ranging from 0 to 1. SR represents straw returning at 4500 kg ha^−1^ with conventional fertilization; BS represents biostimulant foliar application with conventional fertilization; SN, spike numbers; KPS, kernel numbers per spike; TGW, thousand-grain weight; PC, protein content; WGC, wet gluten content; GI, gluten index; SV, sedimentation value; SC, starch content.

Structural equation modeling elucidated the mechanistic pathways through which integrated straw return and biostimulant application enhanced wheat production under saline-alkaline stress (**Figure 8**). The model demonstrated satisfactory fit to the data (χ^2^/df = 2.15, GFI = 0.93, CFI = 0.95, RMSEA = 0.068). Path analysis revealed that SR primarily enhanced soil nutrient supply (β = 0.51, P < 0.001), which subsequently cascaded through improved canopy photosynthetic-nutritional performance (β = 0.63) and aboveground biomass accumulation (β = 0.70 to yield) to increase production. BS demonstrated strong direct effects on canopy photosynthetic-nutritional performance (β = 0.60, P < 0.001), indicating its role as a physiological regulator beyond soil improvement. The strongest pathway to grain yield was through aboveground biomass accumulation (β = 0.70), while grain quality was primarily driven by canopy photosynthetic-nutritional performance (β = 0.62), reflecting distinct regulatory mechanisms for yield and quality formation.

## 4 Discussion

### 4.1 Synergistic mechanisms of straw returning and biostimulant application

The combined application of straw returning and biostimulants demonstrated pronounced synergistic effects that exceeded simple additive responses. The 40.8% yield increase under SR+BS treatment substantially surpassed typical biostimulant efficacy ranges (15-35%) reported in non-stressed environments (Calvo et al., 2014; du Jardin, 2015; Pathak et al., 2024), indicating exceptional synergy under saline-alkaline conditions (pH 8.3, EC 2.84 dS m^−1^). This synergistic enhancement operated through multiple interconnected mechanisms spanning soil-plant system scales.

Biostimulants effectively mitigated the nitrogen immobilization typically associated with straw decomposition. The high C:N ratio of wheat straw initially induced microbial nitrogen immobilization, as evidenced by reduced available nitrogen (58.3 mg kg^−1^) in SR treatment at jointing stage compared to control (71.2 mg kg^−1^). This nitrogen depression represents a critical limitation of straw returning practices (Fan et al., 2024; Chen et al., 2017; Gai et al., 2019; Zhang et al., 2021). However, SR+BS treatment maintained significantly higher available nitrogen (81.5 mg kg^−1^) through dual mechanisms: (1) direct nitrogen supplementation via amino acids (glutamic acid 5 g L^−1^, proline 3 g L^−1^) that bypass microbial immobilization, and (2) enhanced decomposition through Bacillus subtilis-secreted enzymes. The bacterium produces cellulases and hemicellulases that accelerate straw decomposition by 30-50%, effectively shortening the nitrogen immobilization period from 35–40 days to 20–25 days (Hashem et al., 2019; Blake et al., 2021; Ji et al., 2022).

Straw returning reciprocally enhanced biostimulant efficacy by providing carbon substrates and improving soil microenvironments. The 28.3% increase in SOC under SR+BS created favorable conditions for beneficial microorganism activity, as reflected in the 67.9% increase in sucrase activity. This substantial enzyme enhancement indicates accelerated carbon cycling and microbial community activation. Furthermore, organic acids released during decomposition likely reduced rhizosphere pH, improving nutrient availability in alkaline soils. The 47.6% increase in alkaline phosphatase activity under SR+BS, substantially exceeding the increases from individual treatments, suggests improved phosphorus mobilization through synergistic soil-microbe-plant interactions (Dotaniya and Meena, 2015; Dong et al., 2018).

The formation of humic acid-organic matter complexes represented another synergistic mechanism. Humic acid (10 g L^−1^) in biostimulants formed stable complexes with straw-derived organic compounds, reducing carbon mineralization rates while enhancing cation exchange capacity from 12.6 to an estimated 15.8 cmol kg^−1^, improving nutrient retention capacity. These complexes also protected labile carbon fractions from rapid decomposition, extending the duration of beneficial effects on soil properties. The sustained elevation of soil enzyme activities through grain-filling stage under SR+BS treatment, compared to the earlier decline in single treatments, provides evidence for this extended beneficial effect (Canellas et al., 2015; Arslan et al., 2021; Bezuglova et al., 2019).

### 4.2 Nitrogen metabolic transformations and underlying physiological mechanisms

The differential responses of NH_4_^+^-N and NO_3_^−^-N to treatments provide mechanistic insights into nitrogen transformation processes. The 47.7% increase in NH_4_^+^-N under SR+BS treatment reflects accelerated organic nitrogen mineralization, supported by the concurrent 32.1% increase in urease activity. However, the proportionally greater enhancement of NO_3_^−^-N (39.8%) compared to NH_4_^+^-N in SR+BS treatment, coupled with the low NH_4_^+^/NO_3_^−^ ratio (0.41) under BS treatment, indicates rapid nitrification following ammonification. This is consistent with previous findings that Bacillus subtilis-based biostimulants can enhance nitrifier activity through production of extracellular enzymes and modification of rhizosphere pH (Hashem et al., 2019).

The sustained elevation of both nitrogen fractions through grain-filling stage (**Table 2**) ensured continuous nitrogen supply during the critical period of grain protein synthesis. The availability of NO_3_^−^-N is particularly important for wheat quality formation, as nitrate serves as the preferred nitrogen form for long-distance transport and grain loading (Masclaux-Daubresse et al., 2010). The balanced NH_4_^+^/NO_3_^−^ ratio (0.52) maintained under SR+BS treatment may have contributed to the superior grain protein accumulation (16.4%). Both ammonium and nitrate forms can be efficiently assimilated by wheat, with different metabolic costs and uptake kinetics (Lea and Azevedo, 2006; Hawkesford and Griffiths, 2019), and their balanced availability likely optimized nitrogen use efficiency under saline-alkaline stress conditions.

Building upon these soil nitrogen transformations, the substantial improvements in plant nitrogen status under SR+BS treatment provide mechanistic insights into the observed yield and quality enhancement. The 32.1% increase in urease activity, coupled with elevated available nitrogen throughout critical growth stages, indicates enhanced nitrogen cycling capacity in the soil-plant system. Urease catalyzes the hydrolysis of urea to ammonia, representing a rate-limiting step in nitrogen availability (Lea and Azevedo, 2006; Hawkesford and Griffiths, 2019). The sustained high urease activity under SR+BS treatment, maintaining 218 mg NH_4_^+^ kg^−1^ soil h^−1^ at flowering stage, ensured continuous nitrogen supply during periods of peak crop demand.

The enhanced nitrogen assimilation was reflected in superior leaf nitrogen status throughout the growing season. Leaf nitrogen content at grain-filling stage reached 32.7 g kg^−1^ under SR+BS, representing a 45.5% increase over control. This elevated nitrogen status was not merely a result of increased uptake but indicated improved assimilation efficiency, as evidenced by the absence of excessive nitrate accumulation in plant tissues. The optimized nitrogen metabolism was further supported by the coordinated increases in photosynthetic capacity, with chlorophyll content improvements (chlorophyll a: 2.35 mg g^−1^, chlorophyll b: 0.77 mg g^−1^) enabling more efficient light energy capture for nitrogen assimilation processes (Wang et al., 2014; Zheng et al., 2022).

Carbon metabolism optimization complemented the enhanced nitrogen assimilation. The 67.9% increase in sucrase activity under SR+BS treatment indicates substantially accelerated carbohydrate metabolism, providing both energy and carbon skeletons necessary for amino acid synthesis and protein formation. This metabolic acceleration was sustained throughout grain-filling, with soil enzyme activities remaining elevated when control treatments showed declining activity. The maintenance of high chlorophyll content through grain-filling stage, coupled with delayed leaf senescence, extended the photosynthetic active period by approximately 5 days, generating additional photosynthates estimated at 850 kg ha^−1^. This extended photosynthetic capacity provided the carbon resources necessary to support simultaneous increases in both grain yield and protein content (Ruan, 2014; Rangan et al., 2020).

The coordination between carbon and nitrogen metabolism was evident in the improved dry matter accumulation, indicating enhanced dry matter partitioning efficiency to grain. This improvement suggests optimized source-sink relationships, where adequate nitrogen supply stimulated sink capacity development while sustained photosynthetic capacity met the increased sink demand. The foliar application of amino acids during grain-filling likely provided readily assimilable nitrogen forms, reducing the energy cost of nitrogen assimilation and allowing more metabolic resources to be directed toward grain filling (Tegeder and Rentsch, 2010; Huang et al., 2021).

### 4.3 Breaking the yield-quality antagonism through metabolic optimization

The traditional yield-quality tradeoff in wheat production was overcome through optimized carbon-nitrogen balance at multiple biological scales. From a carbon metabolism perspective, SR+BS treatment enhanced photosynthetic capacity through multiple coordinated mechanisms. The 37.5% increase in LAI expanded light interception from approximately 65% to 78% of incident radiation, providing the foundation for increased photosynthate production. The improved chlorophyll content enhanced light harvesting efficiency, while the optimized chlorophyll a/b ratio (3.05-3.12) indicated well-balanced photosystem stoichiometry for efficient energy conversion. Critically, delayed leaf senescence extended the grain-filling period by 5 days, accumulating an additional 850 kg ha^−1^ of photosynthates during this crucial developmental phase (Gregersen et al., 2013; Si et al., 2020; Qin et al., 2024).

Nitrogen metabolism optimization occurred through enhanced uptake, assimilation, and remobilization processes. The 45.5% increase in leaf nitrogen content at grain-filling stage demonstrated superior nitrogen acquisition capacity, supported by the 32.1% increase in soil urease activity that ensured nitrogen availability. The efficient conversion of this elevated nitrogen supply into grain protein, achieving 16.4% protein content (25.2% increase over control), indicates optimized nitrogen partitioning to grain. This efficiency was reflected in the improved nitrogen harvest index, with a greater proportion of plant nitrogen ultimately incorporated into grain protein rather than remaining in vegetative tissues (Masclaux-Daubresse et al., 2010; Liu et al., 2020).

The improved source-sink balance was critical for achieving yield-quality synergy. The grain sink capacity, as indicated by the 17.9% increase in kernels per spike and 11.2% increase in thousand-kernel weight, was more fully utilized under SR+BS treatment. The achievement of high kernel weight (40.8 g) without protein dilution confirmed successful metabolic coordination, where adequate carbon and nitrogen supply met the increased sink demand (Martre et al., 2003; Triboi and Triboi-Blondel, 2002).

### 4.4 Stress mitigation mechanisms under saline-alkaline conditions

Microbial contributions to stress alleviation were substantial and multifaceted. Bacillus subtilis, present at 10^8^ CFU mL^−1^ in the biostimulant formulation, produces exopolysaccharides that form protective biofilms around roots, reducing direct salt contact and improving water retention in the rhizosphere. The bacterium also synthesizes phytohormones including indole-3-acetic acid and gibberellins, which promote root growth and development under stress conditions. The production of ACC deaminase by B. subtilis reduces stress-induced ethylene accumulation, maintaining normal developmental processes and preventing premature senescence. These microbial functions, supported by straw-derived carbon substrates, contributed to the improved root development and nutrient uptake observed under SR+BS treatment (Hashem et al., 2019; Blake et al., 2021; Maslennikova et al., 2023).

The antioxidant system was substantially enhanced under SR+BS treatment, as evidenced by the 44.5% increase in catalase activity. This enzyme increase indicates improved capacity to neutralize hydrogen peroxide and other reactive oxygen species generated under saline-alkaline stress. The coordinated elevation of soil enzyme activities (sucrase, urease, phosphatase, catalase) suggests a more active and stress-resilient soil biological community. This enhanced biological activity likely contributed to improved nutrient cycling and stress alleviation through multiple pathways, including production of stress-protective compounds and maintenance of favorable rhizosphere conditions (Mu et al., 2025).

The soil-level improvements contributed significantly to stress mitigation by creating a more favorable rhizosphere environment. Organic matter accumulation (28.3% increase in SOC) enhanced soil buffering capacity, nutrient retention, and structural properties, facilitating improved root development and function under stress conditions. These soil modifications, combined with enhanced microbial activity and plant physiological adaptations, created a comprehensive multi-level defense system. This integrated approach—simultaneously improving soil conditions, activating beneficial microorganisms, and strengthening plant stress tolerance—explains the exceptional performance under saline-alkaline stress where single interventions typically show limited effectiveness (Munns and Gilliham, 2015; Qadir et al., 2014; Wang et al., 2024).

### 4.5 Field applicability and management guidance for saline-alkaline wheat production

The developed SR+BS system demonstrates strong economic viability and environmental sustainability for saline-alkaline agriculture. Economic analysis reveals treatment costs of 450 yuan ha^−1^ (biostimulant: 300 yuan, straw processing: 150 yuan) against gross returns of 3508 yuan ha^−1^, comprising yield increase benefits (2632 yuan) and quality premium (876 yuan). This achieves a benefit-cost ratio of 7.8:1, substantially exceeding the 3:1 threshold typically required for farmer adoption in China. The additional labor requirements for straw incorporation and biostimulant application (estimated 2–3 person-days ha^−1^) are modest and can be integrated into existing management schedules (Zhang et al., 2024; Shen et al., 2024).

Environmental benefits extend beyond productivity gains and contribute to agricultural sustainability. The system achieves carbon sequestration of approximately 0.85 t C ha^−1^ year^−1^ through increased SOC accumulation, contributing to climate change mitigation while improving soil quality for future production. Nitrogen use efficiency improvement from 32% to 45% reduces environmental nitrogen losses through leaching and gaseous emissions, addressing both water quality and air quality concerns. The productive utilization of agricultural residues (6–8 t ha^−1^ straw annually) addresses waste disposal challenges while providing soil benefits, creating a circular economy approach to resource management. The potential for 20-30% reduction in chemical fertilizer inputs, while maintaining or increasing yields, decreases production costs and reduces the environmental footprint of agricultural production (Smith et al., 2014; Zhao et al., 2018; Fan et al., 2024).

Future optimization strategies should consider several directions for enhancing system performance and adaptability. Biostimulant formulation could be adjusted based on specific soil conditions, with increased humic acid content (12–15 g L^−1^) potentially benefiting severely saline-alkaline soils through enhanced cation exchange and pH buffering capacity, while enhanced amino acid content (8–10 g L^−1^) may address nitrogen-limited conditions more effectively. Application timing optimization using crop growth stage monitoring and stress detection could improve resource use efficiency, with preliminary observations suggesting benefits from split applications at tillering and booting stages in addition to the jointing and grain-filling applications tested here (Yakhin et al., 2017; Pathak et al., 2024).

Straw management refinements offer opportunities for further optimization. Particle size optimization, with preliminary evidence suggesting 2–5 cm as optimal for balancing decomposition rate and soil coverage, could improve both nutrient release kinetics and soil physical property enhancement. Incorporation depth adjustment based on soil type and tillage equipment, with 10–15 cm depths showing promise for balancing surface residue benefits with subsurface organic matter accumulation, warrants systematic investigation. The integration of SR+BS systems with precision agriculture technologies, including remote sensing for stress detection and variable-rate application systems for site-specific management, represents a promising direction for scaling these benefits across heterogeneous field conditions (Daryanto et al., 2017; Si et al., 2020; Mu et al., 2025).

### 4.6 Study limitations and future research directions

While this study provides comprehensive insights into SR+BS synergistic effects on winter wheat production in saline-alkaline soils, several limitations warrant acknowledgment and suggest directions for future research. The two-year experimental duration, though revealing consistent treatment patterns and demonstrating short-term effectiveness, may not capture long-term dynamics of soil organic matter accumulation, potential negative feedback mechanisms, or sustainability of observed benefits. Extended monitoring over 5–10 years is necessary to verify long-term sustainability and identify any diminishing returns or adverse effects. The single soil type (sandy loam) and geographical location, while appropriate for initial mechanism elucidation, limit generalizability across the diverse range of saline-alkaline soils and climatic conditions present in China and globally. Multi-site trials across varying soil textures, salinity levels, and agroecological zones are needed to establish treatment robustness and develop location-specific recommendations (Rouphael and Colla, 2020).

Mechanistic understanding remains incomplete in several critical areas. The specific biochemical pathways through which biostimulant components interact with plant metabolism and stress responses require further elucidation using advanced analytical approaches including metabolomics and proteomics. Rhizosphere microbiome dynamics, including bacterial community composition changes, functional gene abundance, and microbial-plant-soil interactions, need comprehensive characterization through metagenomic sequencing and functional analysis. The fate of straw-derived carbon, including its transformation pathways, stabilization mechanisms, and long-term sequestration potential, warrants investigation using isotope tracing techniques. Understanding these mechanistic details would enable more targeted optimization of biostimulant formulations and application strategies (Bulgari et al., 2019; Blake et al., 2021).

Future research priorities should address several key areas to enhance both fundamental understanding and practical application. Investigation of genotype by treatment interactions could enable development of variety-specific biostimulant formulations that exploit natural genetic variation in stress tolerance and nutrient use efficiency. Evaluation of SR+BS effects under multiple stress scenarios, including heat stress during grain-filling and drought stress at various growth stages, would assess system robustness under climate change conditions. Comprehensive life cycle assessment of environmental impacts, including greenhouse gas emissions, energy inputs, and water use, would provide fuller accounting of sustainability benefits. Socioeconomic analysis of adoption barriers, including farmer perceptions, knowledge requirements, and equipment needs, coupled with policy analysis of institutional and financial support mechanisms, would inform extension strategies and policy development. Integration with digital agriculture platforms for decision support system development, incorporating weather forecasting, crop modeling, and economic optimization, could facilitate broader adoption and adaptive management (Yakhin et al., 2017; Ali et al., 2025; Mu et al., 2025).

## 5 Conclusions

This two-year field experiment demonstrates that integrating straw return with biostimulant foliar application (SR+BS) produces synergistic rather than merely additive effects on winter wheat productivity under mild saline-alkaline stress. The combined treatment resolved the nitrogen immobilization associated with straw decomposition, elevated soil organic carbon and total nitrogen by 28.3% and 19.6%, and markedly enhanced the activities of all four assayed soil enzymes, establishing a more active and stress-resilient soil biological system. These soil-level improvements propagated through enhanced crop physiological performance to achieve simultaneous gains in grain yield (8770 kg ha^−1^, +40.8%) and grain protein content (16.4%, +25.2%), effectively breaking the conventional yield–quality antagonism. Structural equation modeling confirmed a cascade pathway from improved soil carbon-nitrogen supply, through enhanced enzyme activity and inorganic nitrogen availability, to crop productivity — a mechanistic framework with broad relevance for sustainable management of saline-alkaline agroecosystems. While validation across diverse soil types and multi-year timescales is warranted, these findings offer an immediately actionable and economically viable strategy for wheat production on marginal lands.

## Data Availability

The raw data supporting the conclusions of this article will be made available by the authors, without undue reservation.
